# Stomata in Close Contact: The Case of *Pancratium maritimum* L. (Amaryllidaceae)

**DOI:** 10.3390/plants11233377

**Published:** 2022-12-05

**Authors:** Pavlos Saridis, Xenia Georgiadou, Ilana Shtein, John Pouris, Emmanuel Panteris, Sophia Rhizopoulou, Theophanis Constantinidis, Eleni Giannoutsou, Ioannis-Dimosthenis S. Adamakis

**Affiliations:** 1 Section of Botany, Department of Biology, National and Kapodistrian University of Athens, 15784 Athens, Greece; 2 Section of Ecology and Systematics, Department of Biology, National and Kapodistrian University of Athens, 15784 Athens, Greece; 3 Eastern Region Resarch and Development Center, Milken Campus, Ariel 40700, Israel; 4 Department of Botany, School of Biology, Aristotle University of Thessaloniki, 54124 Thessaloniki, Greece

**Keywords:** cell wall, guard cells, homogalacturonans, kidney-shaped, stomata clustering

## Abstract

A special feature found in Amaryllidaceae is that some guard cells of the neighboring stomata form a “connection strand” between their dorsal cell walls. In the present work, this strand was studied in terms of both its composition and its effect on the morphology and function of the stomata in *Pancratium maritimum* L. leaves. The structure of stomata and their connection strand were studied by light and transmission electron microscopy. FM 4–64 and aniline blue staining and application of tannic acid were performed to detect cell membranes, callose, and pectins, respectively. A plasmolysis experiment was also performed. The composition of the connection strand was analyzed by fluorescence microscopy after immunostaining with several cell-wall-related antibodies, while pectinase treatment was applied to confirm the presence of pectins in the connection strand. To examine the effect of this connection on stomatal function, several morphological characteristics (width, length, size, pore aperture, stomatal distance, and cell size of the intermediate pavement cell) were studied. It is suggested that the connecting strand consists of cell wall material laid through the middle of the intermediate pavement cell adjoining the two stomata. These cell wall strands are mainly comprised of pectins, and crystalline cellulose and extensins were also present. Connected stomata do not open like the single stomata do, indicating that the connection strand could also affect stomatal function. This trait is common to other Amaryllidaceae representatives.

## 1. Introduction

Stomatal complexes connect plants with the atmosphere, controlling dispersal of gases and harmonizing carbon dioxide uptake with water vapor loss [1]. Therefore, regulation of their development is critical to enable plants to adjust gas exchange in the presence of environmental challenges [2]. Their distribution on leaf surfaces is a tightly orchestrated process, but shows a level of plasticity [3]. Stomata ontogenesis has been comprehensively studied, and factors controlling the cellular processes involved are constantly identified [1]. The “typical” monocot leaf epidermis consists of long, axially oriented pavement cells, interspersed with much shorter stomata, either alone (anomocytic) or associated with small lateral subsidiary cells (LSCs) (paracytic–nonoblique) [4,5]. The “one-cell spacing” rule, (i.e., stomata are separated by at least one cell), is a typical example of cellular “spacing pattern” and has long intrigued developmental biologists [3,6,7,8,9,10]. Τhe one-cell spacing rule is crucially important to the gas-exchange capacity of leaves (in both dicots and monocots). In addition to its effect on gas diffusion, there are obvious impediments to the function of guard cells themselves when this rule is broken [11,12]. In angiosperms, the stomatal pore is created by the guard cells bending apart from each other and at the same time exerting tremendous force on the cell walls of the adjacent epidermal cells [8]. When stomata clustering occurs or the “one-cell spacing” rule is defied in any way, the biomechanics of stomata are heavily affected and they cannot open properly. However, little is known yet on the limitations clustering places on the normal operation of stomata [13].

The Amaryllidaceae family includes approximately 850 species found in diverse ecosystems widely distributed in temperate and tropical regions (South America, southern Africa, and the Mediterranean) [14]. *Pancratium maritimum* L., also known as sea daffodil, is a bulbous perennial plant of Amaryllidaceae, and its name reveals its habitat, since the adjective “maritimum” means “of the sea” and is a typical plant species on sandy shores (sand dunes), spreading from the Mediterranean to the Black Sea, including the coasts bordering the Atlantic Ocean [15]. The environmental conditions that exist in this specific habitat are extremely harsh: exposure to wind from the sea, high salinity, reduced availability of nutrients and fresh water, high exposure to radiation, high temperatures, soil instability, and sandblasting [16,17]. However, *P. maritimum* has developed specific physiological mechanisms to overcome this harsh environment [18,19]. Its pavement cells are polygonal and elongated with oblique or straight anticlinal walls. Dispersed anomocytic kidney-shaped stomata occur on both leaf epidermises (adaxial, abaxial) [20]. During anomocytic stomata development, the meristemoid develops to a guard cell mother cell, which divides anticlinally to form two equal guard cells, gradually becoming kidney-shaped [21]. Specifically, in *P. maritimum* leaf stomata are arranged lengthwise on intercostal areas of the epidermises. Stomata are slightly submerged, and usually alternate with pavement cells. Guard cells are kidney-shaped and sometimes carry waxes just like epidermal cells. The stomatal pore appears to be elongated and the ventral cell wall displays thickening [20].

The principal works examining anatomy in the Amaryllidaceae were performed using several species from diverse countries of the world [22,23,24]. These authors report the internal structure of Amaryllidaceae species belonging to several species and genera. The connection strands between the guard cells of two neighboring stomata are an interesting feature, encountered in the leaves of *Amaryllis* spp., *Crinum asiaticum* and *Pancratium* spp., all representatives of the Amaryllidaceae family. It has been reported [25], that these connections are protrusions emerging from one of the adjacent cells of the stomata, growing until they finally meet and fuse with the neighboring stoma. It has been hypothesized that the cell wall decomposes at the point of contact and a communication channel develops between the two neighboring stomata. These connections have been described as cytoplasmic protrusions of guard cells, like plasmodesmata. In this study, it was considered rather intriguing to examine in detail the composition and morphology of this connection, as well as its frequency of occurrence. In addition, the effect of this connection on the functionality of the stomata was addressed. Moreover, the occurrence of this feature was studied in some Amaryllidaceae species from diverse environments and parts of the world.

## 2. Results

### 2.1. Morphological and Anatomical Features of Pancratium maritimum Leaf

**Figure 1:**
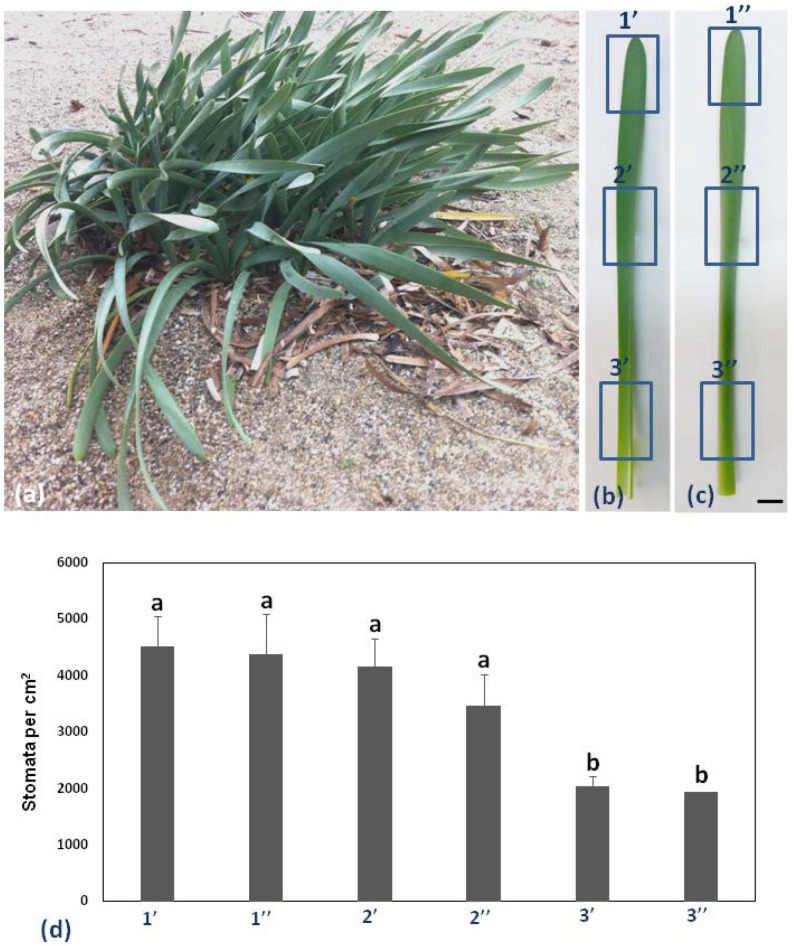
*Pancratium maritimum* morphology and stomata density. (**a**) *P. maritimum* plants growing in a sand dune. b, c: The six parts of the leaf blade [1′, 2′, 3′ abaxial (lower), 1′, 2′, 3′ adaxial (upper)]. (**b**) The adaxial surface and (**c**) the abaxial surface of the same leaf. (**d**) Diagram of stomata distribution in six parts of the leaf of *P. maritimum*. Measurements are the mean of 10 leaves of the same size. Statistical significance (*p* < 0.05) is marked by the different letters. Scale bars in b, c: 1 cm.

*Pancratium maritimum* leaves are broadly linear and glaucous as shown in Figure 1a. Six different regions (adaxial top, middle, and base, 1′, 2′, and 3′, respectively, Figure 1b, abaxial top, middle, and base, 1′, 2′, and 3′, respectively, Figure 1c, of the same leaf) of the leaf on 10 different leaves, were chosen to measure stomatal complex density per cm^2^ (Figure 1d).

**Figure 2:**
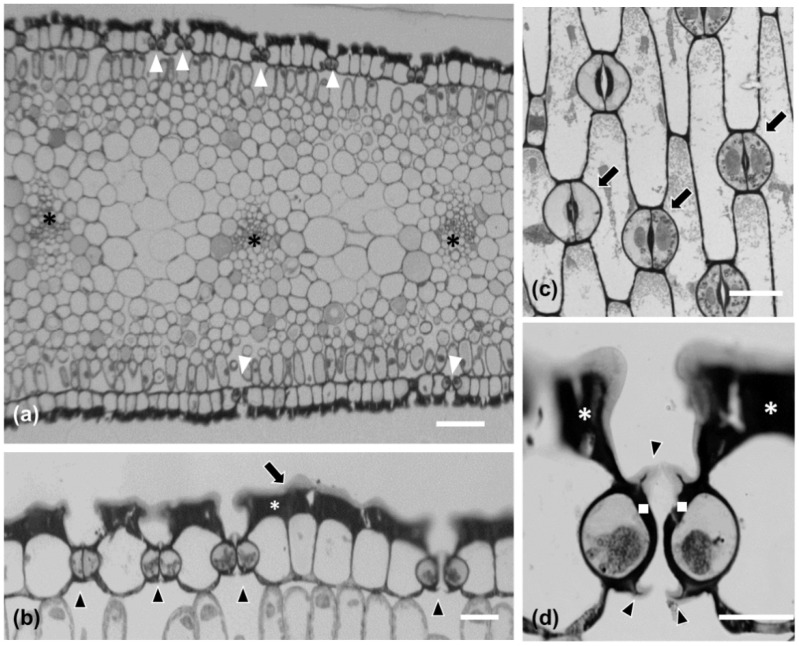
Leaf (**a,b**) and stomatal (**c,d**) morphological and anatomical characteristics in *Pancratium maritimum*. (**a,b,d**) Transverse sections after toluidine staining of resin-embedded material. (**a**) The leaf is amphistomatic [white arrows in (**a**)], while vascular bundles are located in the middle of the mesophyll [asterisks in (**a**)]. (**b**) Higher magnification of leaf epidermis. Leaf epidermis is covered by a thick cuticle [long arrow in (**b**)]. Arrowheads point to stomata, while white asterisk shows the thick periclinal external cell wall of the epidermal cells. (**c**) Paradermal section of the leaf where anomocytic stomata (arrows) and pavement cells are shown. (**d**) Transverse section of a closed stoma where the cell wall thickenings of the ventral cell wall (white squares), as well as the ledges (arrowheads) are shown; the white asterisks depict epidermal periclinal cell wall thickenings. Scale bars: 20 μm.

Stomatal density appeared to decrease in a basipetal direction, while it was lower on the abaxial than the adaxial epidermis (Figure 1d). *P. maritimum* leaf epidermis is covered by a thick cuticle (arrow in Figure 2b) and is amphistomatic (arrowheads in Figure 2b). Individual vascular bundles (asterisks in Figure 2a) were observed in the middle of the mesophyll. Guard cells were kidney-shaped (arrows in Figure 2c). No special subsidiary cells were found flanking guard cells (anomocytic stomata) (arrows in Figure 2c). Guard cells display thickenings in their ventral cell wall (white squares in Figure 2d), while ledges are formed on both sides (arrowheads in Figure 2d). Pavement cells display a particularly thickened external periclinal cell wall (white asterisks in Figure 2b,d), while they appear rectangular on cross-section (Figure 2a,b) and their long axis is parallel to that of the leaf (Figure 2c).

### 2.2. Fine Structure of Pancratium maritimum Guard Cells

**Figure 3:**
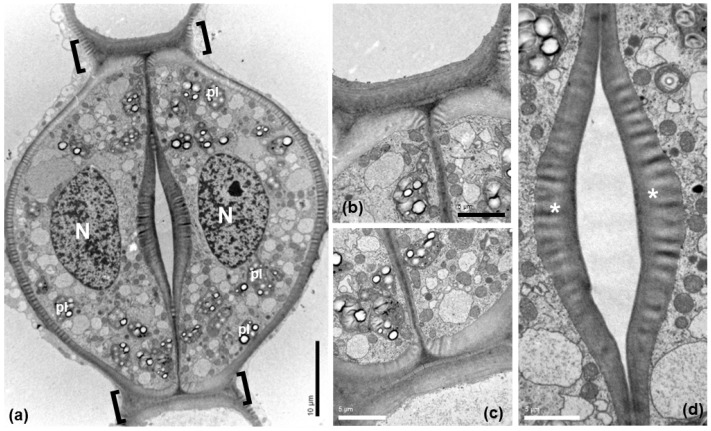
Transmission electron microscopy (TEM) depicting a stoma of *Pancratium maritimum* leaf at paradermal section. (**a**) Low magnification presenting guard cell fine structure. (**b**,**c**). The transverse cell wall ends, defined by brackets in (**a**), are thickened. (**d**) Ventral walls are thickened at the site of the stomatal pore. Note the non-uniform electron density of the thickenings at the areas denoted by asterisks. N: nucleus, pl: plastid. Scale bars as depicted on image.

The guard cells of mature *Pancratium maritimum* stomata (Figure 3) include large nuclei (N in Figure 3a), starch-rich plastids (pl) and numerous mitochondria. Prominent thickenings were observed (Figure 3a–c) at the transverse guard cell wall ends, at the junctions of the dorsal with the ventral cell walls. The ventral cell walls were also locally thickened (Figure 3a,d), exhibiting a particular morphology (asterisks in 3d).

### 2.3. Neighboring Stomata Are Often Connected by a Strand

**Figure 4:**
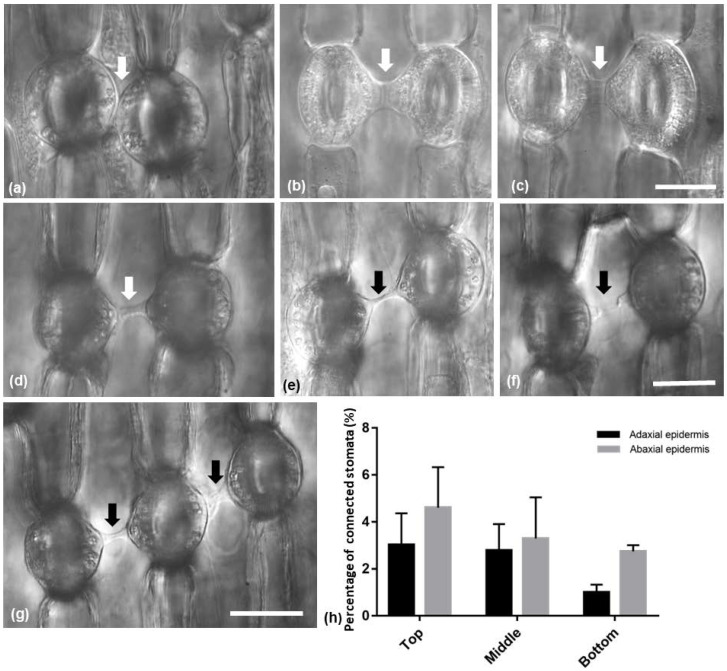
(**a**–**g**) Light micrographs of paradermal leaf sections with DIC (Nomarski) optics, showing (arrows) the morphological variety among the observed connecting strands of adjacent stomata. (**h**) Diagram showing the percentage of connected stomata on the adaxial and the abaxial epidermis. The values are means of ten different leaves of the same size; significant differences (*p* < 0.05) were not found. Scale bars: 20 μm.

In both *Pancratium maritimum* leaf epidermises, stomata connected to each other by a specific strand were observed (Figure 4). The connecting strands varied in length and/or width (Figure 4a–g) or even appeared broken (Figure 4f). Attached stomata were present (Figure 4a). Sometimes, three stomata in a row appeared to be connected by such strands (Figure 4g). Stomata appeared also to be connected with epidermal cells and meristemoids (Figure S1). The percentage of stomata that were connected by a strand decreased towards the base of the leaf and was higher on the abaxial than the adaxial epidermis; however, no statistically significant differences were detected (Figure 4h).

### 2.4. Morphology and Fine Structure of Connecting Strands

**Figure 5:**
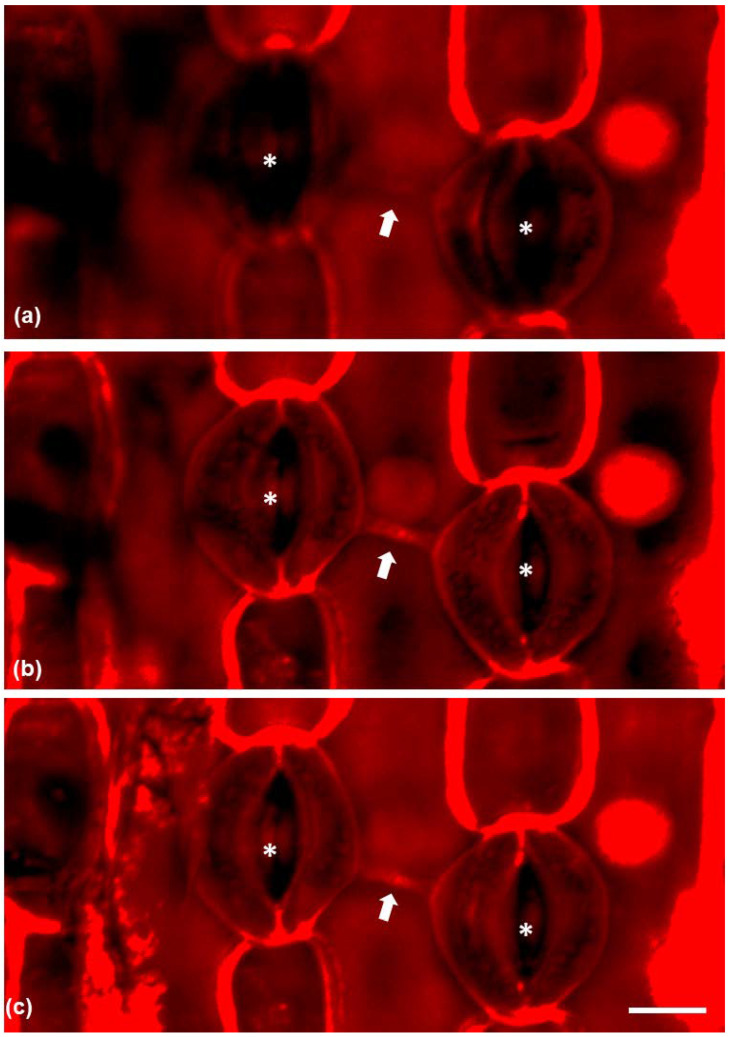
CLSM micrographs of epidermal peels stained with propidium iodide, depicting connected stomata (asterisks) and the connection strand between them (arrow). (**a**) CLSM section on the external region of the epidermal cell between the stomata. (**b**) A median CLSM section and (**c**) CLSM maximum projection. Scale bar: 20 μm.

Propidium iodine-stained paradermal sections were observed under CLSM (confocal laser-scanning microscopy) (Figure 5) in order to visualize the connection between neighboring stomata. The connection strand was positively stained by propidium iodine and series of CLSM sections revealed that the connection was located under the external periclinal cell wall of the epidermal cell between the connected stomata (Figure 5a–c) as it was clearly visible in median CLSM sections and not at external ones.

**Figure 6:**
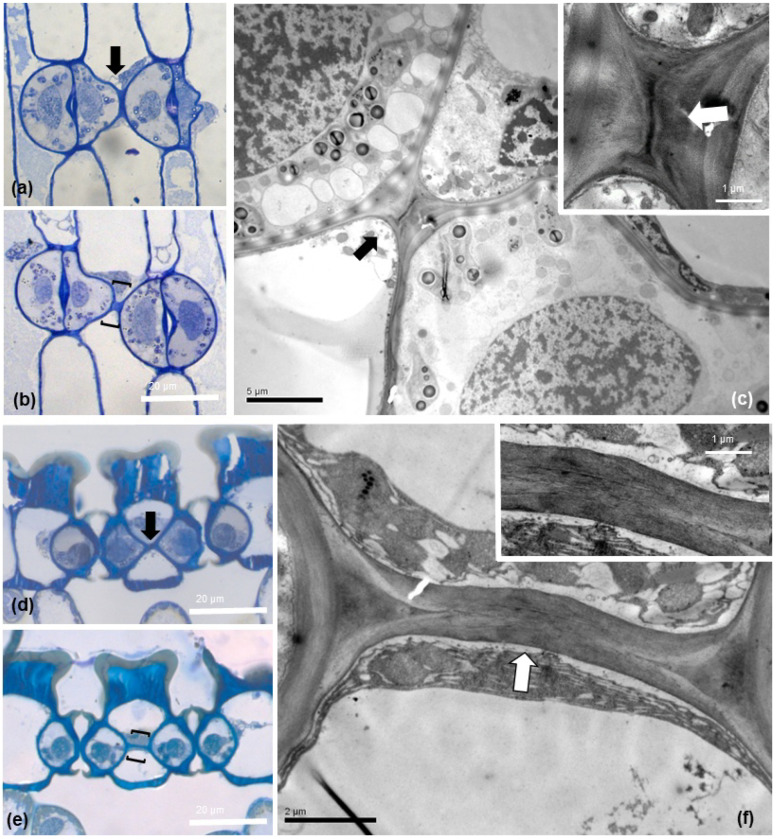
Light microscopy (**a**,**b**,**d**,**e**); toluidine blue O staining) and TEM (**c**,**f**) micrographs of paradermal (**a**–**c**) and transverse (**d**–**f**) sections of *Pancratium maritimum* leaves. The connection strand is indicated either by arrows (**a**,**c**,**d**,**f**) or brackets (**b**,**e**). In TEM micrographs (**c**,**f**) the cell wall texture of the depicted connections between adjacent stomata is identified. Insets show the areas indicated by the arrows at higher magnification. Scale bar as depicted on images.

Observation of semithin sections by light microscopy (Figure 6a,b,d,e) revealed that the connecting strand appears as a cell wall projection, not of cytoplasmic nature. Examination of the areas included in brackets in 6b and 6e by TEM specified the cell wall structure of this cell wall connection strand, either inhomogeneous, (Figure 6c) or homogeneous (Figure 6f), implying a spatial heterogeneity in the cell wall composition of the specific structure.

### 2.5. Composition of the Strand Connecting Adjacent Stomata

**Figure 7:**
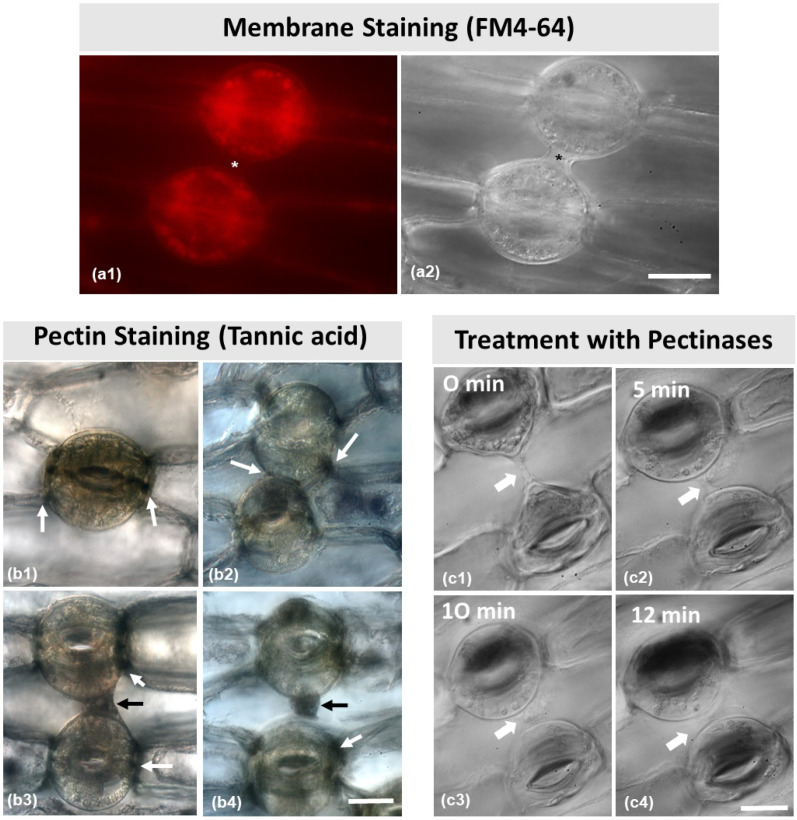
(**a**) Membrane staining with FM 4–64 (**a1**). No signal can be observed in the structure connecting the dorsal cell walls (asterisks in **a1**,**a2**) of two adjacent stomata (also imaged with DIC optics in (**a2**). (**b**) Tannic acid–ferric chloride staining for localization of pectins. Arrows point to pectin localization. Pectins are located at the junction sites of the dorsal cell walls with the neighboring epidermal cell (**b1**), at the junction sites of the dorsal cell walls of the adjacent stomata (**b2**), and at the strand connecting the dorsal cell walls of two adjacent stomata (**b3**,**b4**). (**c**) Consecutive photographs (**c1**–**c4**) of a pair of adjacent connected stomata during treatment with 3% pectinase solution (treatment duration as noted on each figure). The connection between the stomata gradually disappears. Scale bars = 20 μm.

The fluorescent dye FM 4–64 is widely used to identify membrane components in the cell. After staining (Figure 7(a1)), it was observed that the connection strand (asterisks in Figure 7(a1,a2)) between the two stomata did not emit any fluorescent signal.

As shown in Figure 7(b1–b4), the strand material was positive to tannic acid staining, which specifically reacts with pectic materials in the cell wall. As pointed to by the white arrows in Figure 7(b1–b4), pectins were present in the junction sites of the cell walls between guard cells, as well as between the dorsal cell walls and the adjacent epidermal cells. Furthermore, as pointed to by the black arrows, pectins were located at the cell wall strands depicted in Figure 7(b3,b4). 

**Figure 8:**
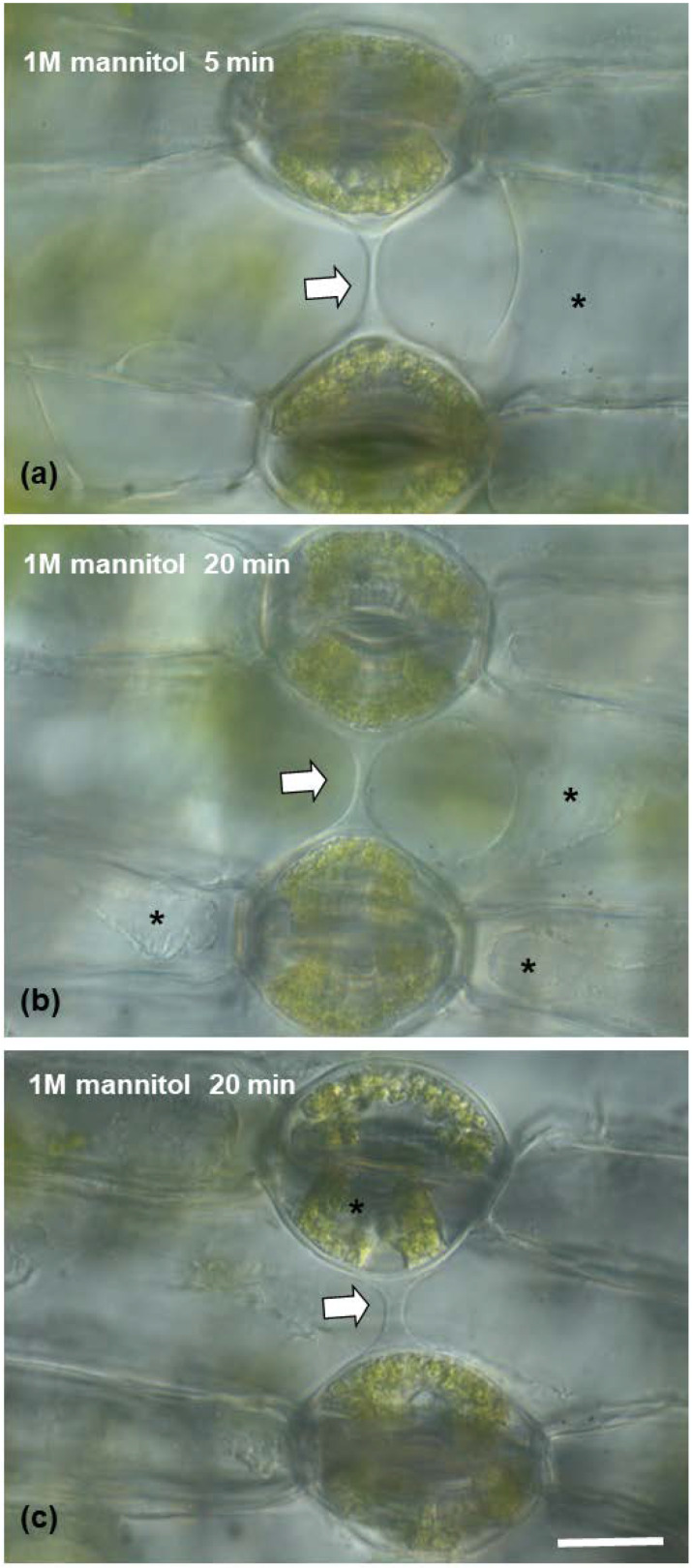
(**a**–**c**) Light micrographs of epidermal peels with DIC (Nomarski) optics, showing the connection strand (arrow), which retains its structural integrity in the presence of a hypertonic 1 M mannitol aqueous solution after 5 (**a**) or 20 (**b**,**c**) minutes. The connection strand is present, while the cytoplasm of the epidermal (**a**,**b**) or guard cells (**c**) appears plasmolyzed (asterisks). Scale bar: 20 μm.

After treatment with 3% pectinase (Figure 7(c1–c4)), the connection strand appeared to diminish and gradually disappear, starting from 5 min treatment to 12 min (Figure 7(c2–c4)), which confirms that the strand consists mainly of pectins. When epidermal peels were subjected to 1 M of mannitol aqueous solution (Figure 8) for either 5 min or 20 min (Figure 8a–c), the connection strand was not affected, while the cytoplasm of the epidermal and guard cells was plasmolyzed.

### 2.6. Cell Wall Matrix Material Detection at the Connection Strand

**Figure 9:**
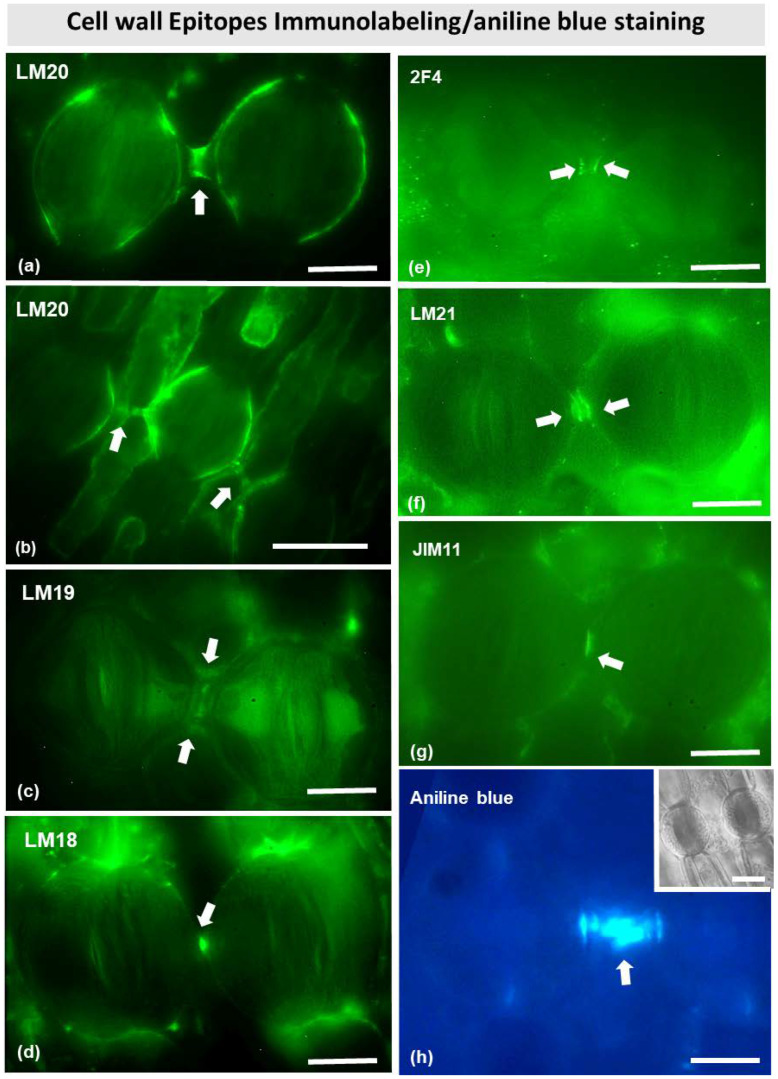
Immunolabeling of cell wall epitopes (**a**–**g**) and aniline blue fluorescent staining (**h**) in stomata of *Pancratium maritimum* epidermis. In all panels, arrows point to sites with intense fluorescent signal. (**a**,**b**) LM20 (fully methylesterified homogalacturonans) immunolabeling of two (**a**) and three (**b**) adjacent stomata. When the connecting strand is relatively short (**a**), the LM20 signal is detected at the dorsal cell walls and at the middle of the strand. When it is relatively long (**b**), it seems absent from the middle of the cell wall strand. (**c**–**e**) LM19, LM18 and 2F4 immunolocalization (unesterified homogalacturonans, partially demethylesterified homogalacturonans, and partially demethylesterified homogalacturonans with 40% calcium bridges, respectively) immunolabeling of two adjacent stomata. LM19 and LM18 signal is detected at the middle of the connecting strand (**c**,**d**) while 2F4 is located at the junctions of the strand with the dorsal cell walls (**e**). (**f**) LM21 (mannans) immunolabeling of two adjacent stomata. The signal is detected at the dorsal cell walls and at the middle of the connecting strand. (**g**) JIM11 (extensins) immunolabeling of two adjacent stomata, where the signal is detected at the connecting strand. (**h**) Aniline blue staining provides intense signal at the connecting strand. Inset: representative stomata in DIC. Scale bars: 10 μm.

Immunostaining with several antibodies specific for various cell wall matrix epitopes (see Materials and Methods) allowed the identification of cell wall components (Figure 9) in the stomata of *Pancratium maritimum*. Immunodetection with LM20 antibody revealed that the connection strand, as well as the dorsal cell walls (Figure 9a,b) are rich in fully methylesterified homogalacturonans. The fluorescent signal was very intense at the center of the strand, where electron-dense material was found by TEM (Figure 6c). Apart from methylesterified homogalacturonans, the connection strand also comprised partially demethylesterified and unesterified homogalacturonans, as can be observed after immunolabeling with LM18 and LM19 antibodies (Figure 9c,d). Interestingly, the epitopes detected by 2F4 antibody (Figure 9e), which corresponds to demethylesterified homogalacturonans connected with calcium bridges, were found at the site where the cell wall strand meets the dorsal cell walls of the neighboring guard cells.

Immunostaining of mannans with the LM21 antibody revealed their localization at the junctions of the dorsal cell walls with the adjacent epidermal cells (Figure 9f), while a strong signal was detected at the junctions of the connection strand with the dorsal cell walls. Furthermore, intense fluorescent signal was observed also with the JIM11 antibody, which detects extensins. The signal was located at the junction sites of the dorsal cell walls with the neighboring epidermal cells, as well as at the connection strand between two adjacent cells (Figure 9g). Aniline blue fluorescence also confirmed the presence of callose in the connection strand (Figure 9h).

### 2.7. High Crystalline Cellulose Content in the Connecting Strands

**Figure 10:**
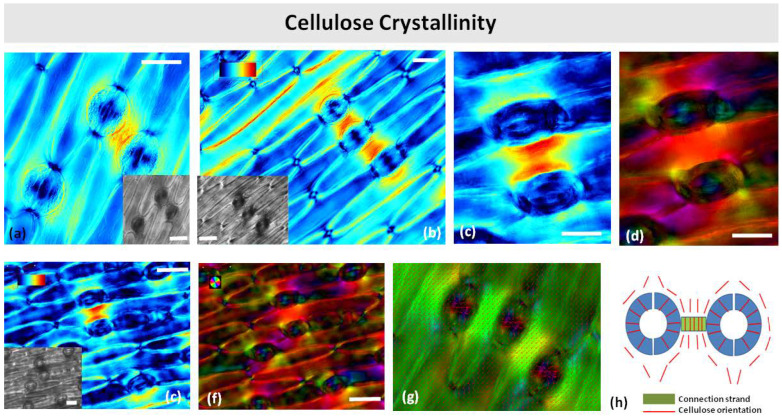
Polarized light color-coded PolScope images of crystalline cellulose retardance (crystallinity) (**a**–**c**,**e**) and orientation (**d**,**f**,**g**) in paradermal sections of *Pancratium maritimum* leaves. (**a**,**b**) Stomata with connection strands by DIC optics (insets in (**a**,**b**,**e)**) and cellulose retardance images (**a**–**c**,**e**). (**h**) Schematic representation of cellulose orientation in a stomatal pair with connection strand. Orientation scale color codes the cellulose microfibrils’ orientation. Retardance scale color codes the retardance range (0–120 nm). Scale bars: 20 μm.

Polarized light microscopy revealed that the connection strands were more retardant than the surrounding epidermis or regions near other stomata (Figure 10a,b,e,f). Apparently, the strands contain more crystalline cellulose. As the strands were observed under a relatively high retardance range (0–120 nm), the retardance pattern on the guard cell walls was not visible. The cellulose microfibril orientation in the stomatal guard cells followed the typical radial pattern, while microfibril orientation in the surrounding pavement cells was parallel to the long axis of the cell (Figure 10c,d). In the cell wall strands, the cellulose microfibrils were oriented perpendicularly to the long axis of the strand, i.e., parallel to the leaf axis (Figure 10c,d,h).

### 2.8. The Cell Wall Connection Strand Seriously Affects Guard Cell Shape and Pore Aperture

**Figure 11:**
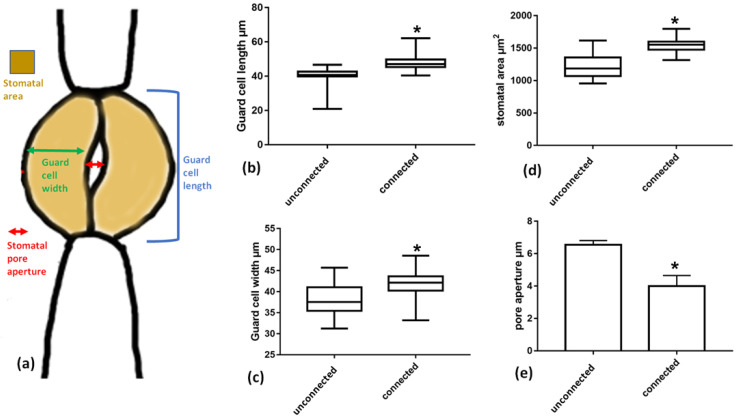
Comparison of dimensions between connected vs. unconnected stomata as defined in a stomatal schematics (**a**). (**b**) Guard cell length, (**c**) guard cell width, (**d**) stomatal area, (**e**) stomatal pore aperture. Asterisks mark statistically significant differences (*p* < 0.05). The presented values are mean measurements from 100 stomata.

To investigate the possible impact of this strand connection on the morphology and shape of guard cells, the dimensions of guard cells of stomata connected with a cell wall strand were measured and compared to those of unconnected stomata. It was found that guard cell length (blue line in Figure 11a) and width (green line in Figure 11a) differs between connected and unconnected stomata. Guard cells of connected stomata are longer and wider than those of unconnected stomata, while the whole stomatal area is significantly higher in connected stomata. Importantly, although connected stomata are larger, the stomatal pore aperture (red line in Figure 11a) is smaller in the connected stomata than in unconnected ones.

### 2.9. The Connection Strand Limits the Expansion of the Intervening Pavement Cell

**Figure 12:**
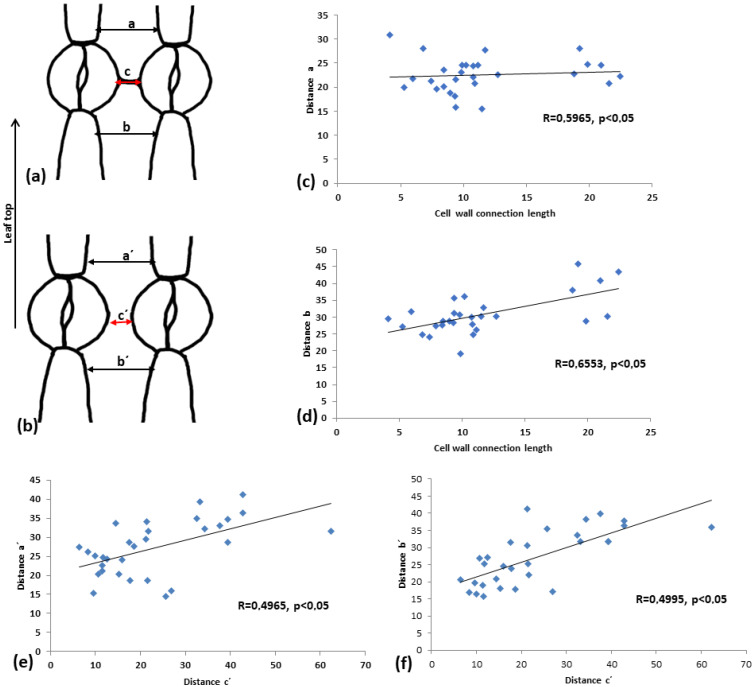
Comparison of the distance, as shown in (**a**,**b**), between connected vs. unconnected adjacent stomata. (**c**,**d**) Diagrams for connected adjacent stomata, (**e**,**f**) diagrams for unconnected adjacent stomata. (**c**,**e**) Diagrams showing the correlation between the distance c (cell wall connection length, in the case of connected stomata) to the distance a. (**e**,**f**) Correlations between distances a’ and b’ to distance c’. Every result is statistically significant at *p* < 0.05, and the calculated R coefficient is depicted on the graphs. Mean values from 100 stomata pairs are presented.

To assess how this cell wall structure may affect the shape of the intervening pavement cell, pavement cell width was measured at the side towards leaf tip (a and a’) and towards leaf base (b and b’). In addition, the distance between adjacent stomata (c and c’) was measured (see drawings in Figure 12a,b). In the linear correlation diagrams (Figure 12c–f), the correlations of the above measurements are depicted. It can be observed that when connection strands are present (Figure 12c,d), the coefficient of linear correlation (R) is higher, implying a strong correlation between the two parameters tested.

### 2.10. The Cell Wall Strand Is a Universal Feature Present in Various Subfamilies of Amaryllidaceae

**Figure 13:**
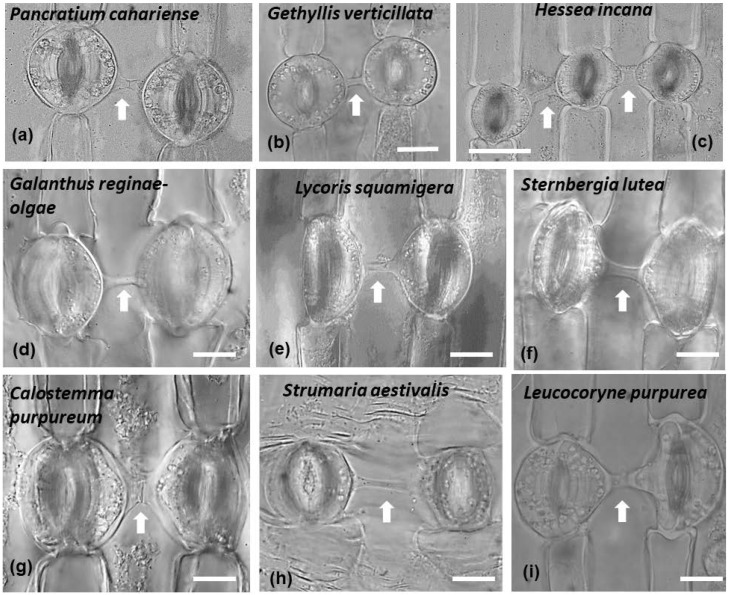
Connected stomata in leaves of representative species (the name is given on each panel) of the three subfamilies of Amaryllidaceae: Amaryllidoideae, Allioideae and Agapanthoideae. Stomata appear connected in pairs (**a**,**b**,**d**–**i**) and sometimes triplets exist (**c**). Arrows point to cell wall connection strands. Scale bar: 20 μm.

Connected stomata in connection are generally not a common feature of plant epidermises. In all the species studied, it was noticed that connected stomata were found almost exclusively where a single pavement cell was located between them (Figure 4 and Figure 13). Some stomata were connected diagonally, while connected stomata appeared usually in pairs. Sometimes triplets of connected stomata could be observed as well (Figure 4c and Figure 13c).

## 3. Discussion

The phenomenon of connected stomata, as well as other stomatal peculiarities, were extensively studied, in the epidermis of several plants [22]. Species of the genera *Asparagus*, *Asphodelus*, *Allium* of the Asparagaceae, Asphodelaceae and Amaryllidaceae families were examined. Also, a wider range of species was examined [24], though the focus was on the Amaryllidaceae family. The considered species were *Agapanthus umbellatus, Allium cepa, Allium sativa, Allium tuberosum, Amaryllis belladonna, Amaryllis yittate, Nerine curvifolia, Crinum zeylanicum, Crinum asiatisum, Cyrtanthus mackenii, Zephyranthes candida, Cooperia pedunculata, Haemanthus multiflorus, Pancratium verecundum* and *Narcissus tazetta.* In addition, connected stomata (with the percentage of connected stomata to vary among the species) have been identified in *Narcissus pseudonarcissus, Amaryllis reticulata, Crocus sativus, Ixiolirion tataricum,* and *Iris* Langport Wren, while other morphological peculiarities of stomata were also found in *Amaryllis reticulatα*, *Narcissus pseudonarcissus*, *Iris* Langport Wren, *Crocus sativus*, and *Ixiolirion tataricum* but not in *Allium cepa* [25]. In accordance, we observed connected stomata in the species *Hessea incana, Strumaria aestivalis, Gethyllis verticillata, Haemanthus albiflos, Galanthus reginae-olgae, Pancratium canariense, Sternbergia lutea, Leucocoryne purpurea* and *Pancratium maritimum*, upon which we focused our research.

No matter the species, the connection strand is a structure that brings together two neighboring guard cells of two (or even three) adjacent stomata. The connection observed between the two stomata crosses the cytoplasm of the intervening pavement cell approximately in the middle of the distance between its periclinal cell walls (brackets in Figure 6e). Though until now this connection has been characterized as “cytoplasmic,” [20,22,24,25], the findings of this study reveal that this connection does not consist of any membranous material (i.e., no staining with FM 4.64 or no effect on it when epidermal peels were subjected to a hypertonic solution) and should be considered as a cell wall strand. This cell wall strand contains fully and partially methylesterified homogalacturonans, homogalacturonans with low levels of methylesterification associated with each other by calcium bridges, and nonesterified homogalacturonans (LM20, LM18, 2F4, LM19 respectively). The endings of the cell wall strand that connect it to the middle of the dorsal cell walls of the guard cell contain partially methylesterified pectins and pectins with methylesterification levels up to 40% (LM18 and 2F4, respectively), while the middle of the strand contains mainly fully methylesterified homogalacturonans (LM20) and to a lesser extent nonesterified homogalacturonans (LM19). In addition, it contains hemicelluloses, namely, callose (along the entire length of the junction) and mannans (LM21), as well as extensins (JIM11) (along the entire length of the binding and mainly in its periphery). The homogalacturonan (HG) domain of the pectic network is implicated in affecting many of the cell wall properties that impact cell expansion, development, intercellular adhesion, and defense mechanisms [26]. Since LM20 was intensively present in this cell wall strand, highly methylesterified HG polysaccharides that are deposited in the cell wall can potentially be modified in different ways to generate distinct polysaccharides with diverse functional properties [27] present in this specific cell wall structure. It can be concluded that this connection is quite flexible in its middle, which allows it to expand and become thinner (relatively thin connection strands seem to be devoid of LM20 signal in their middle part) and eventually break up, as stomata are set further apart following leaf expansion and elongation. Furthermore, this structure is strongly stabilized at the points of its binding with the dorsal guard cell walls of neighboring stomata, given the physical properties that each of the aforementioned cell wall component provide to the cell wall [28,29].

In the model plant *Arabidopsis thaliana*, mutations (*tmm*, *flp* etc.) of genes that orchestrate stomatal ontogenesis induce intense stomatal clustering [30], but environmental conditions that limit gas exchange from the atmosphere also lead to the same effect (i.e., plants grown in a closed flask) [11]. Various environmental parameters, such as light intensity, atmospheric CO_2_ concentration, water availability [31], or even immersion of the plants in 1–5% sucrose solution [32], affect stomatal density. Thus, stomatal aggregations are affected by both environmental and genetic parameters [33,34,35,36]. Stomatal clustering has also been observed in *Begonia*, where it has been considered an adaptation for growth in arid environments [37]. In the Amaryllidaceae family, a relationship of connected stomata linked to the presence to environmental conditions is highly unlikely, since they were present both in plants living in sandy seashores (i.e., *Pancratium* species [38]), and in plants grown in open woodland, woodland, damp and shady spaces (i.e., *Sternbergia lutea* and *Galanthus regine-olgae* [39]). We may therefore conclude that it is a rather typical characteristic for the leaf epidermis of Amaryllidaceae and not correlated with the ability of the plants to survive in arid environments [40]. Therefore, this unique characteristic is highly unlikely to be an adaptation to specific environmental conditions. On the contrary, this feature does not seem to provide any advantage, but very probably hampers the stomatal ability to open widely.

When stomatal clusters are present, stomatal proximity affects their ability to change their shape, possibly altering their stable connection to their adjacent epidermal cells, thus affecting expansion [13]. In *Pancratium maritimum* the size of intervening pavement cells between connected stomata is affected by the presence of the connecting cell wall strand and cannot expand as much as in the absence of the strand. This is in accordance with high cellulose crystallinity, detected at the site of the strand. Although cellulose microfibril orientation does not seem to limit the expansion of the intervening pavement cell, the presence of high crystalline cellulose at the specific site restricts its expansion [41] and possibly hinders the ability of the stoma to properly open when needed. Indeed, stomatal pore aperture appeared decreased in connected stomata. This implies an impact of the connection strand in the opening and closing of stoma. It is possible that the specific structure causes a deformity in the cell shape and the guard cells lose their ability to modify their shape accordingly to meet the needs required each specific moment. Another factor affecting stomata opening is calcium ion concentration [42]. In order to keep their osmotic pressure high, guard cells take up amounts of calcium from the surrounding epidermal cells. It is possible that stomatal aggregation limits the access to this source and prevents opening [12]. However, in our work, because this connection strand occupied a small area of the whole guard cell surface, calcium uptake hampering is very unlikely to be responsible for the observed defects.

Stomata follow a “one-cell spacing” rule by which two stomata are separated by at least one intervening epidermal cell [43]. Disturbance of this rule in *P. maritimum* and other Amaryllidaceae representatives was found in naturally occurring plants and is not derived by any kind of genetic manipulation. The genetics of the Amaryllidaceae family remain largely unknown [44]. It has been suggested [22] that connected stomata occur when two meristemoids are connected. This would probably be true, since we observed connected guard mother cells and stomata connected to meristemoids or epidermal cells (Figure S1). In addition, since the connection strand consists principally of pectins, it could probably originate from the middle lamella of adjacent cells, which is also composed of pectins [45]. It could be speculated that the genetic machinery, that orchestrates stomatal “one-spacing” rule, malfunctions in Amaryllidaceae, resulting to the occurrence of those peculiarly connected stomata. However, plants usually achieve to correct such “mistakes,” and eventually stomata break apart. The exact developmental mechanism behind this “nature’s caprice” could be investigated in the future. In this work, it has been shown that cell wall biomechanics play a crucial role in stomatal development and proper function, as has already been stated in previous works [46,47,48]. Violating the one-cell spacing rule, not only by stomatal clustering but also by a cell wall strand connecting adjacent stomata, seems to influence guard cell shape and possibly its function. Further investigation on the successive steps of connection strand formation is required, in order to improve our understanding of its role in the various Amaryllidaceae plants in which it was detected.

## 4. Materials and Methods

### 4.1. Plant Material

*Pancratium maritimum* plants were collected from the sandy beach zone of the areas of Potamos, Epanomi, Thermaikos Gulf, Greece (40°22′56″ N, 22°55′37″ E), Mourteris, Evia Island, Greece (38°37′16.8″ N, 24°07′14.1″ E) and Rishon LeTsiyon, Israel (31°58′5.6″ N, 34°43′39.6″ E). *Sternbergia lutea* and *Galanthus reginae-olgae* plants were collected from Imittos Aesthetic Forest, Attika, Greece (37°57′56.3″ N, 23°47′93.2″ E) and Hortiatis Mountain, Thessaloniki, Greece (40°35′04″ N, 23°07′22″ E), respectively. The plants were maintained in a mixture of sand and peat, or peat alone in a controlled growth chamber at 25 °C in a 12 h light/12 h dark cycle [19]. Other representatives of the Amaryllidaceae (*Hessea incana*, *Strumaria aestivalis*, *Gethyllis verticillata*, *Haemanthus albiflos*, *Pancratium canariense*, *Leucocoryne purpurea*) were supplied from cultivated collections (cordially provided by E Dardiotis).

#### Chemicals

All chemicals and reagents were purchased from Applichem (Darmstadt, Germany), Merck (Darmstadt, Germany), PolySciences (Niles, IL, USA), SERVA (Heidelberg, Germany) and Sigma-Aldrich (Taufkirchen, Germany) and all the procedures described below were performed in triplicate at room temperature, unless otherwise stated.

### 4.2. Methodology

#### 4.2.1. Light Microscopy

The light microscopy observations, except polarized light, were made using a Zeiss Axioplan (Carl Zeiss AG) microscope, equipped with a UV source and a differential interference contrast (DIC) optical system, a Zeiss Axiocam MRc5 digital camera and appropriate filters. Imaging was achieved with ZEN 2.0 software according to the manufacturer’s instructions and digital images were processed with Adobe Photoshop CC 2015 with only linear settings. Some specimens were also observed with a Zeiss Observer Z1 inverted microscope equipped with LSM780 CLSM module and ZEN 2011 software.

##### Measurement of Stomatal Density in Various Leaf Areas

Ten different leaves from about ten different plants were used. In each, hand-cut paradermal sections were cut at six areas: apex, middle and base of the adaxial epidermis and at the corresponding sites of the abaxial epidermis. Each section was measured with a ruler and its area calculated. Then, measurement of the different types of stomata (single stomata, stomata in connection with each other) and calculation of their percentage in the various areas was performed by light microscopy.

##### Measurement of the Morphological Characteristics of the Neighboring Stomata

Paradermal sections from the top of the adaxial epidermis of *Pancratium maritimum* leaves were cut and observed with light microscopy. Several stomatal anatomical features (length and width of the guard cells, length, and width of the intervening epidermal cell between two adjacent stomata, distance between two neighboring stomata) were measured. The total stomatal area was also measured in μm^2^, assuming the shape of the stoma as an ellipse (π × r1 × r2) such that the stomatal area equals to π (length/2) × (width/2).

##### Stomatal Pore Opening

Leaf pieces were placed in wet filter paper and placed in a chamber at 30 °C for about 20 min. The sections were then cut into smaller ones and were observed under a light microscope. Measurements of stomatal characteristics such as pore opening, stomatal complex length, and cell width were made. Stomatal width was then calculated, where it equals the opening of the stomatal pore plus the width of the two guard cells of the stomatal complex.

#### 4.2.2. Experimental Procedures

##### Aniline Blue Stain

A solution of 0.05% aniline blue in 0.07 M K_2_HPO_4_ buffer, pH 8.5, was used [49] to detect the presence of callose. Paradermal handmade leaf sections were mounted directly on slides with a drop of aniline blue and observed under a fluorescence microscope at a wavelength of 455 nm.

##### FM 4–64 stain

Paradermal hand-cut leaf sections were immersed for 10 min in a 5 μM FM 4–64 aqueous solution (Invitrogen, Carlsbad, CA, USA), which was diluted from a 10 mM stock solution in DMSO. After 2–3 washes with distilled water, the sections were placed on slides for microscopic observation at an appropriate wavelength [50].

##### Tannic Acid–Ferric Chloride Stain

The specific staining reveals the pectins [51]. Initially, incubation in a 1% tannic acid solution in distilled water was carried out for 10 min. After 10 min washes in distilled water, the sections were incubated in a 3% aqueous solution of iron chloride (FeCl_2_) for 10 min. After three washes in distilled water, the sections were observed under a light microscope.

##### Propidium Iodide Staining

An aqueous propidium iodide (Sigma) solution (1 mg/mL for 5 min, at room temperature) was used to stain epidermal cells and stomata in peeled leaf epidermis [52]. Propidium iodide shows an affinity for pectin [52]. The specimens were afterwards observed with CLSM.

##### Pectinase Treatment

Paradermal sections were cut and placed on a glass slide in a drop of water and then covered with a coverslip. An area containing connected stomata was then selected for observation. The water in the slide was replaced by 3% or 6% pectinase (pectinase from *Aspergillus niger*; Merck) solution in PBS. Pectinase’s effect was recorded by micrograph and video under a light microscope equipped with differential interference contrast (DIC) optics.

##### Mannitol Treatment

In order to verify that no cytoplasmic material is involved in the connection strand, epidermal peels were also subjected to 1 M mannitol aqueous hypertonic solution to induce plasmolysis [53]. Epidermal peels were observed after 5 and 20 min under a light microscope equipped with differential interference contrast (DIC) optics.

##### Cell Wall Material Immunolocalization

In order to observe the cell wall matrix materials, immunolocalization with various antibodies was carried out. First, removal of the cuticle was performed [54], either with a depilatory tape (peeling method), and then processed as follows:

For immunolabeling of LM20, LM19, LM18, 2F4, JIM11, LM21 HG epitopes in epidermal handmade leaf sections, the protocol [55] in handmade leaf sections was used modified accordingly. LM20, LM19, LM18, 2F4, JIM11, LM21 (Plant Probes, Leeds, UK) were used as primary antibodies and FITC-conjugated anti-rat IgG (Sigma) as secondary antibody in all cases. Among various HG epitopes, the distribution of HGs with a high degree of esterification recognized by LM20 antibody [56], HGs with a low degree of esterification recognized by LM19 and LM18 antibody [52] and Ca^2+^ cross linked HGs with a low degree of esterification recognized by 2F4 antibody [57] has been studied. All antibodies were diluted 1:40 in PBS that contained 2% (*w*/*v*) BSA except for 2F4 and its secondary antibody, which were diluted 1:40 in T/Ca/S buffer (Tris-HCl 20 mM pH 8.2, CaCl_2_ 0.5 mM, NaCl 150 mM). During the immunolabeling procedure with 2F4 antibody, the sections were washed with T/Ca/S buffer.

##### Sample Preparation for Transmission Electron Microscopy (TEM)

Leaf pieces 3 × 3 mm^2^ were fixed in 3% GA (glutaraldehyde) + 2% paraformaldehyde in 50 mM sodium cacodylate buffer, pH 7, at room temperature for 3 h. After 2 washes, 10 min each, with buffer, the samples were postfixed in 1% (*w*/*v*) osmium tetroxide (OsO_4_) for 3 h at room temperature. This was followed by 3 washes of 15 min each with buffer. The samples were afterwards dehydrated in an acetone series, treated with propylene oxide and placed in a mixture of Spurr’s resin and propylene oxide (1:3 v/v) overnight for embedding. The samples were gradually transferred in increasing resin content mixtures and finally in pure resin. Finally, the samples were placed in pure resin at 65 °C for 3 d (72 h) for polymerization. Semithin sections (1.5–2 μm) were cut with a glass knife and stained with 1% toluidine blue O and observed by light microscopy. Ultrathin sections (70–90 nm) were cut with a diamond knife and double-stained with 2% uranyl acetate in 70% ethanol and 1% Reynolds lead citrate and observed at 80 kV with a JEOL JEM 1011 (JEOL, Tokyo, Japan) TEM, equipped with a Gatan ES500W digital camera. Digital images were recorded with Gatan Digital Micrograph software. Figures obtained were optimized for contrast and brightness with the Adobe Photoshop CS6 software with only linear settings.

##### Polarized Light Microscopy

Crystalline cellulose is a strongly birefringent material. The LC-PolScope image processing system (CRi, Inc., Woburn, WA, USA) equipped with liquid crystal (LC) compensator enables in situ determination of the sample light retardance, from which cellulose crystallinity and orientation can be assessed [58,59]. PolScope was mounted on a light microscope (Nicon Eclipse 80i, Tokyo, Japan) equipped with a cooled CCD camera. The stomata were observed on epidermal peels from the abaxial side of five fully expanded mature leaves, with retardance range of 0–120 nm. The image focus was chosen by comparing visible light, orientation and retardance images.

##### Statistical Analysis

Wherever necessary to compare the mean values between two different experimental groups, the paired *t*-test method was applied. Those showing *p* < 0.05 were considered statistically significant results. The *t*-test analysis was done using the GraphPad Prism version 4.0 (San Diego, CA, USA).

## 5. Conclusions

According to our data, the structure connecting the dorsal cell walls of two adjacent stomata is formed at the middle of the intermediate pavement cell adjoining the two stomata. It is comprised of cell wall matrix materials, with various homogalacturonans and callose to be the main components and is prone to pectinase treatment. Cellulose crystallinity is increased in the epidermal cell wall at the site where the two stomata are connected by a strand. Furthermore, this structure seems to influence the shape of the stomata and the size of their aperture, possibly affecting normal stomatal function. These cell wall strands seem to influence also the intermediate epidermal cell width. Cell wall connection strands between neighboring stomata were observed in the three subfamilies of the Amaryllidaceae family, namely, Amaryllidoideae, Allioideae and Agapanthoideae.

## Data Availability

Not applicable.

## Supplementary Materials

The following supporting information can be downloaded at https://www.mdpi.com/article/10.3390/plants11233377/s1. Figure S1: Light micrographs of paradermal leaf sections with DIC (Nomarski) optics, showing the variety of the stomata peculiarities observed in *Pancratium maritimum*.
