# Supplementary material for: Stomata in Close Contact: The Case of Pancratium maritimum L. (Amaryllidaceae)
Source: Plants (Basel). 2022 Dec 5;11(23):3377. doi: 10.3390/plants11233377 (PMC9740904; doi:10.3390/plants11233377)
Supplement: Supplementary file 1 [file plants-11-03377-s001.zip › plants-2018823-supplementary.pdf]

Stomata in close contact: The case of *Pancratium maritimum* L. (Amaryllidaceae)

Pavlos Saridis [1], Xenia Georgiadou [1,2†], Ilana Shtein [3], John Pouris [1], Emmanuel Panteris [4], Sophia Rhizopoulou [1], Theophanis Constantinidis [2], Eleni Giannoutsou [1*], Ioannis – Dimosthenis S. Adamakis [1*]

1 Section of Botany, Department of Biology, National and Kapodistrian University of Athens, 15784 Athens, Greece
2 Section of Ecology and Systematics, Department of Biology, National and Kapodistrian University of Athens, 15784 Athens, Greece
3 Eastern R&D Center, Milken Campus, Ariel, Israel
4 Department of Botany, School of Biology, Aristotle University of Thessaloniki, 541 24 Thessaloniki, Greece
† Current address: Institute of Natural Sciences, University of Graz, NAWI Graz, Heinrichstrasse 28, Graz 8010, Austria
* Correspondence: EG: egianno@biol.uoa.gr , I-D. S. A: iadamaki@biol.uoa.gr

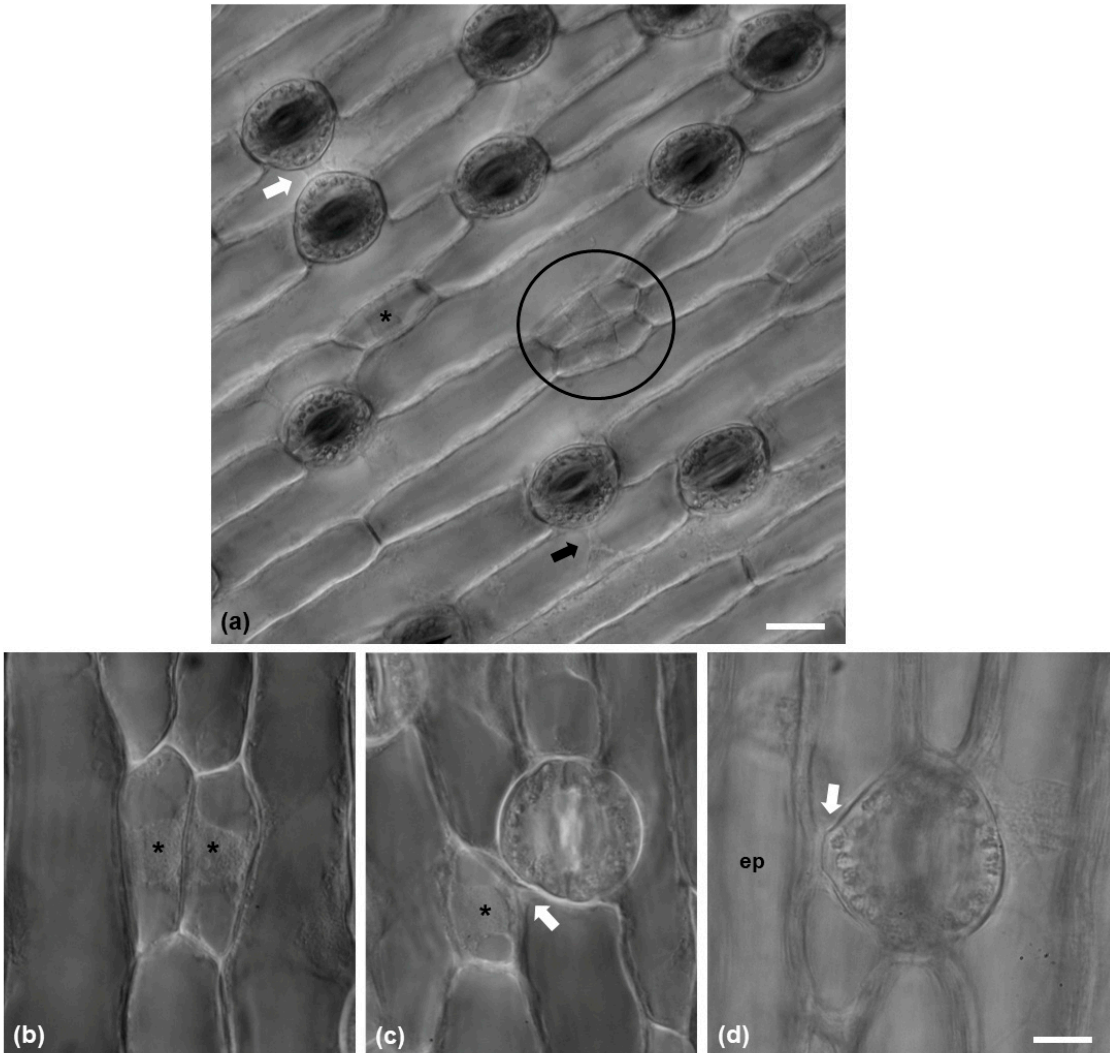

Figure S1. (a-d) Light micrographs of paradermal leaf sections with DIC (Nomarski) optics, showing the variety of the stomata peculiarities observed in *Pancratium maritimum*. (a) Overview of an epidermal area where connected stomata (white arrow), a single guard mother cell (asterisk), two guard mother cells in contact (circle) and a stomata connected to an epidermal cell (black arrow) are depicted. (b) A higher magnification of the guard mother cells (asterisks) in contact is shown in (a). (c) A meristemoid (asterisk) in contact (arrow) with a stoma. (d) A stoma connected (arrow) with an epidermal cell. Ep: epidermal cell. Scale bar in (a): 10 μm. Scale bars in (b, c, d): 20 μm.
